# Paracrine Role of the Endothelium in Metabolic Homeostasis in Health and Nutrient Excess

**DOI:** 10.3389/fcvm.2022.882923

**Published:** 2022-04-26

**Authors:** Cheukyau Luk, Natalie J. Haywood, Katherine I. Bridge, Mark T. Kearney

**Affiliations:** Leeds Institute of Cardiovascular and Metabolic Medicine, Faculty of Medicine and Health, University of Leeds, Leeds, United Kingdom

**Keywords:** endothelium, adipose tissue, paracrine signaling, cardiometabolic syndrome, obesity, metabolism, endothelial dysfunction, adipose tissue dysfunction

## Abstract

The vascular endothelium traditionally viewed as a simple physical barrier between the circulation and tissue is now well-established as a key organ mediating whole organism homeostasis by release of a portfolio of anti-inflammatory and pro-inflammatory vasoactive molecules. Healthy endothelium releases anti-inflammatory signaling molecules such as nitric oxide and prostacyclin; in contrast, diseased endothelium secretes pro-inflammatory signals such as reactive oxygen species, endothelin-1 and tumor necrosis factor-alpha (TNFα). Endothelial dysfunction, which has now been identified as a hallmark of different components of the cardiometabolic syndrome including obesity, type 2 diabetes and hypertension, initiates and drives the progression of tissue damage in these disorders. Recently it has become apparent that, in addition to vasoactive molecules, the vascular endothelium has the potential to secrete a diverse range of small molecules and proteins mediating metabolic processes in adipose tissue (AT), liver, skeletal muscle and the pancreas. AT plays a pivotal role in orchestrating whole-body energy homeostasis and AT dysfunction, characterized by local and systemic inflammation, is central to the metabolic complications of obesity. Thus, understanding and targeting the crosstalk between the endothelium and AT may generate novel therapeutic opportunities for the cardiometabolic syndrome. Here, we provide an overview of the role of the endothelial secretome in controlling the function of AT. The endothelial-derived metabolic regulatory factors are grouped and discussed based on their physical properties and their downstream signaling effects. In addition, we focus on the therapeutic potential of these regulatory factors in treating cardiometabolic syndrome, and discuss areas of future study of potential translatable and clinical significance. The vascular endothelium is emerging as an important paracrine/endocrine organ that secretes regulatory factors in response to nutritional and environmental cues. Endothelial dysfunction may result in imbalanced secretion of these regulatory factors and contribute to the progression of AT and whole body metabolic dysfunction. As the vascular endothelium is the first responder to local nutritional changes and adipocyte-derived signals, future work elucidating the changes in the endothelial secretome is crucial to improve our understanding of the pathophysiology of cardiometabolic disease, and in aiding our development of new therapeutic strategies to treat and prevent cardiometabolic syndrome.

## Introduction

Cardiometabolic syndrome (CMS), or metabolic syndrome, is characterized by a combination of metabolic dysfunction, including hyperglycaemia, insulin resistance, hyperlipidaemia, obesity and hypertension. The global prevalence of CMS is continuing to rise and, in 2018 it was estimated to affect 25% of the population (1). To address the differences in clinical definitions of the disease by various health organizations, the International Diabetes Federation proposed the latest accepted worldwide definition of CMS; central obesity and two of following: elevated triglycerides, lower HDL(high-density lipoprotein)-cholesterol, elevated blood pressure and elevated fasting plasma glucose level (2). These metabolic and cardiovascular perturbations individually and in combination, contribute to a substantial increase in cardiovascular disease morbidity and mortality.

Many environmental factors can contribute to the onset and development of central obesity in CMS, notably recent changes in human lifestyle, such as increased sedentary behaviors and consumption of high calorific foods (1). Opposing the conventional view that obesity is purely a metabolic disorder, the concept of “immunometabolism” describes the previously underdetermined relationship between chronic inflammation in metabolic organs and the brain, and energy imbalance in obesity (3). Several pioneering papers revealing the role of inflammation in metabolic organs, and its link to metabolic defects, focussed on the interaction between macrophages and adipocytes in adipose tissue (4–6). The idea of immunometabolism was then explored and expanded further into other organs including liver, skeletal muscle, pancreas, gastrointestinal tract and brain (3, 7). In addition, endothelial dysfunction is believed to play an important role in the pathophysiology of CMS, as it is well-established that obesity and metabolic dysfunction are able to disrupt a number of signaling pathways in the endothelium (8). With specific attention to interactions between the vascular endothelium and adipocytes, we review the role of endothelial-derived factors in mediating metabolic and inflammatory signaling in adipose tissue (AT), both in the context of normal physiology, and in the pathophysiology associated with CMS.

### Endothelial Dysfunction in CMS and the Emerging Interest of Endothelial Secretome in Health and Disease

The endothelium, anatomically defined as the inner cellular lining of blood and lymphatic vessels, acts as a physiological barrier, which prevents direct contact between tissues and the circulation (9). It also plays a role in mediating angiogenesis, inflammation, thrombosis, vascular tone and energy homeostasis (8–12). Endothelial dysfunction is often defined as the reduced production or availability of nitric oxide (NO), the elevated release of vasoconstrictors such as endothelin-1 (13) and the upregulation of cell adhesion molecules, associated with enhanced leukocyte adherence, increased cell permeability, and platelet activation (14). Endothelial dysfunction is a well-established response to cardiovascular risk factors and precedes the development of atherosclerosis (15).

The endothelium is a dynamic organ, and is one of the first responders to metabolic and vascular changes (16), such as insulin level (17) and blood flow (18). Due to its intimate relationship with nearby cells, impaired endothelial barrier function, or imbalanced endothelial secretion of metabolites, results in dysregulated signaling pathways of peripheral tissues, which in turn contributes to the development of metabolic dysfunction and chronic tissue inflammation. Time-course experiments on diet-induced obese mice revealed the preceding role of endothelial dysfunction in the development of metabolic dysfunction and chronic inflammation in liver, muscle and AT (19). In obesity, dysfunctional endothelium displays a more pro-inflammatory and pro-fibrotic phenotype, resulting in increased vascular leakage and acceleration of tissue inflammation by recruitment and activation of immune cells (20).

In addition to acting as a physical barrier between local tissues and the circulation, the endothelium plays an important role in signaling. Since the discovery of endothelial-derived relaxing factor (EDRF), now identified as NO, the endothelial secretory profile has gained much attention (21, 22). There is a growing body of evidence of the presence of endothelial-derived small membrane vesicles, which are released to the extracellular space (23). These findings further establish the existence of an endothelial secretome and raise the question of its role in various diseases including obesity in CMS.

### Adipose Dysfunction and Its Treatment Options in Obesity

Obesity is a multisystem disorder driven by nutrient excess, leading to perturbation of glucose homeostasis, immune dysregulation, dyslipidaemia and disadvantageous alterations in adipocyte phenotype, which leads to a vicious cycle of cellular dysfunction contributing to CMS (15, 24). During nutrient excess, white AT (WAT) undergoes expansive remodeling, whereby white adipocytes adopt a hypertrophic/hyperplastic phenotype (25), accompanied by neovascularisation to support this remodeling (26, 27). In advanced obesity there is a mismatch between WAT expansion and vascularisation leading to inadequate perfusion (28), adipocyte ischaemia (28), inflammation (28–30), secretion of pro-inflammatory mediators (31) and the requirement to store lipids in tissues ill-equipped to deal with this challenge, all contributing to CMS (32).

AT is crucial in storing excess energy and maintaining thermal homeostasis (33). It is dynamically regulated by a wide range of physiological cues, including norepinephrine, insulin and acetylcholine (33). There are two main types of AT; WAT which is specialized for the storage of energy in the form of triglyceride (34) and thermogenic brown AT (BAT), which unlike WAT expresses uncoupling protein-1 (UCP-1) (35). UCP-1 uncouples cellular respiration from mitochondrial ATP synthesis, affording thermogenic AT the capacity to burn fat and generate heat (36). Recent studies indicate that at least two distinct types of thermogenic adipocyte exist in mammals: a pre-existing form established during development, termed classical BAT, and an inducible form, “beige” adipocytes. Beige adipocyte biogenesis can be stimulated by various environmental cues, such as chronic cold exposure, in a process frequently referred to as “beiging” of white fat (37, 38). The thermogenic capacity of beige adipocytes and the possibility of controlling their expression has stimulated substantial attention due to its potential application in the mitigation of obesity-related disease (39–42). Although extensive efforts have been made pharmacologically to activate AT thermogenesis, these attempts have been unsuccessful due to poor bioavailability and concerns over the side effects of the agents used (43). More importantly, a recent study reported that human brown adipocyte thermogenesis is actually mediated by β_2_-adrenoceptors, instead of β_3_-adrenoceptors as in rodents (44), explaining the lack of efficacy of β_3_-adrenoceptor agonists to activate brown adipose thermogenesis or white adipose beiging. Thus, identifying alternative pathways to promote beige adipose biogenesis could lead to therapeutic interventions which reduce cardiovascular risk.

Alternative therapeutic goals also include reducing AT inflammation and fibrosis (45). Under normal physiology, inflammation is important in maintaining healthy adipogenesis and adipocyte function and helps regulate whole-body glucose tolerance (46). However, chronic adipocyte inflammation can link to metabolic defects such as insulin resistance (46–48). Current therapeutics targeting AT inflammation do not always present positive outcomes in human trials and potential risk of increased susceptibility to infections is yet to be explored (45). On the other hand, we still await clinical studies employing anti-fibrotic agents to determine the efficacy and safety of targeting WAT fibrosis to treat CMS.

### Scope of Review

AT is highly vascularized with a capillary network, allowing the reciprocal transport of nutrients, substrates and oxygen. During AT expansion, the capillary network also expands to meet the increasing demand for nutrients and oxygen (49). With particular interest in dissecting the communication between endothelial cells and adipocytes, here we review the role of endothelial-derived molecules in mediating metabolism and inflammation in the pathophysiology of CMS. We focus on the synthesis, release and the paracrine action of the endothelial secretome in AT and discuss how it may be exploited therapeutically in the future. Endothelial-derived molecules are grouped according to their physical properties.

## Gaseous Regulators

### Nitric Oxide (NO)

Endothelial-derived NO has been traditionally viewed as a key modulator of cardiovascular function, and reduced bioavailability of NO has been associated with inflammation and vascular disease (10, 50). More recently, data suggests that NO also acts as a metabolic modulator, and reduced circulating NO levels have been observed before the development of peripheral insulin resistance in diet-induced obesity (12, 19). Under normal physiological conditions, endothelial nitric oxide synthase (eNOS, also known as NOS3) is active and generates NO transiently. The activity of eNOS is regulated by shear stress within vessels. The synthesis of NO can also be elevated upon stimulation by endogenous signaling molecules such as insulin, insulin-like growth factor 1 (IGF-1), acetylcholine and the pro-inflammatory molecule bradykinin (51–54). Within AT, eNOS-mediated NO generation can be upregulated by adipocyte-secreted adiponectin (55) and leptin (56). Apart from its well-known vasodilatory effect via activating the soluble guanylate cyclase (sGC)-generated cyclic guanosine monophosphate (cGMP) pathway in vascular smooth muscle, NO is thought to be important in regulating oxygen transport and mitochondrial respiration in other tissues (57). In contrast, in diseases such as CMS, eNOS activity is suppressed whereas inducible NOS (iNOS) in the endothelium is activated to generate potentially pathophysiological concentrations of NO, which reacts with superoxide to form peroxynitrite (58). Peroxynitrite is cytotoxic and is a known pro-atherosclerotic factor (58, 59) (reviewed in Peroxynitrite (ONOO^–^)).

A range of factors, including elevated plasma levels of free fatty acids, pro-inflammatory cytokines such as tumor necrosis factor-alpha (TNFα), oxidized low-density lipoprotein (OxLDL) and asymmetric dimethylarginine, all common features of CMS, are known to inhibit eNOS activity (60–63). Under prolonged high fat diet, mice display profoundly reduced eNOS expression, specifically in AT suggesting diseased adiposity is coupled with a decline in eNOS activity (64). The WAT of diet-induced obese mice demonstrates a reduction in NO coupled with increased AT inflammation, characterized by increased gene expression of the pro-inflammatory TNFα (65). eNOS-deficient mice are insulin-resistant and hyperlipidaemic (66, 67), even on a chow diet, AT from eNOS-deficient mice displays a notably inflammatory profile, characterized by increased gene expression of TNFα, interleukin-6 (IL-6) and monocyte chemoattractant protein-1 (MCP-1) (65). Together these findings suggested endothelial-derived NO is insulin-sensitizing and anti-inflammatory in AT under normal physiological conditions. Promisingly, in eNOS-deficient mice, the addition of dietary nitrate at a dose just enough to restore physiological NO levels, is able to reverse the unfavorable metabolic sequelae, reducing both visceral fat deposition and circulating triglycerides (68). Restoring NO downstream signaling by administration of sildenafil also reduces AT inflammation in high fat diet-challenged mice (65). Complementary studies using mice overexpressing eNOS, demonstrate that eNOS over-expression offers protection against diet-induced obesity, with a more metabolically active AT, characterized by smaller adipocyte size and elevated mitochondrial biogenesis and metabolism (64). Taken together these murine studies show that eNOS activity is important in regulating AT function and inflammation.

Various therapeutic approaches have been employed to determine whether targeting NO can improve AT metabolism in rodents and humans. S-nitrosoacetyl penicillamine (SNAP) an NO donor has been shown to enhance mitochondrial biogenesis in primary murine brown adipocyte precursors and cultured 3T3-L1 white adipocytes, in a process mediated by cGMP (69). Nitrate, which can be utilized to produce NO under hypoxia, has been shown to have beneficial effects in AT in several studies. Nitrate can induce gene expression of beiging markers in cultured primary murine white adipocytes (70, 71), as well as inducing other favorable metabolic changes, including upregulation of mitochondrial respiration, increased uptake and oxidation of fatty acid and glucose (71, 72). Application of nitrite on palmitate-treated primary mouse white adipocytes also induces beiging (73). Although eNOS is not the only source of NO in mammals due to the presence of other isoforms of NOS and dietary nitrate intake, these findings support the hypothesis that eNOS-derived NO promotes adipocyte beiging and mitochondrial biogenesis.

eNOS-derived NO has also been well-characterized as an anti-inflammatory mediator. It inhibits platelet aggregation (74) and acts on the endothelium to suppress expression of adhesion molecules, thus suppressing leukocyte recruitment and accumulation (75). Impaired eNOS-NO signaling may contribute to AT inflammation in obesity, as characterized by increased leukocyte adhesion and platelet aggregation (76).

In rodent models, dietary nitrate resulted in beiging of white AT in rats (71) and more interestingly, in mice challenged with high fat diet, dietary nitrate supplementation reduced weight gain, improved glucose tolerance and attenuated AT inflammation (73, 77). Chronic dietary supplementation of L-arginine (the precursor of NO) reduced weight gain, circulating glucose and triglyceride levels in Zucker diabetic fatty rats (78). Human studies have also confirmed the benefits of targeting NO in CMS. In a randomized, double-blind, placebo-controlled clinical trial, 6 weeks of dietary L-arginine supplementation reduced central adiposity in women with obesity (79). In another clinical trial, 6 months of dietary L-arginine supplementation improved whole body insulin-sensitivity in individuals with obesity (80). However, conflicting results showing lack of efficacy of L-arginine treatment have also been reported in studies of patients with obesity and insulin resistance, raising the doubt of therapeutic potential of L-arginine (81, 82). A recent systematic review and meta-analysis of clinical trials suggested L-arginine supplementation resulted in reduced waist circumference but had no effect in reducing body weight or BMI (83). Future studies determining the appropriate dosage and length of the treatment are needed to improve treatment outcome using L-arginine.

## Proteins Mediators

### Endothelin-1 (ET-1)

Endothelin was the first potent endothelium-derived vasoconstrictor to be identified. ET-1 was isolated from the culture media of porcine aortic endothelial cells as a 21 amino acid peptide (84). Two other endothelins, ET-2 and ET-3, were later identified. The endothelin family is synthesized and released upon cleavage of its precursors by endothelin-converting enzymes (85). The synthesis of ET-1 can be upregulated by endogenous mediators such as thrombin (84), angiotensin II (86), insulin (87), lipoproteins (88, 89), and suppressed by NO (90) and prostacyclin (91). Secreted endothelin acts on endothelin receptors type A and B (ET_A_R and ET_B_R) and mediate glucose and lipid metabolism in the AT (92).

In *in vitro* experiments using mature 3T3-L1 adipocytes, ET-1 upregulated glycerol release in a dose-dependent manner (93). This effect was prevented by pre-treatment using an ET_A_R antagonist BQ-610, but not an ET_B_R antagonist BQ-788, suggesting ET-1-induced lipolysis was mediated by ET_A_R (93). In time-course experiments the effect of ET-1 on glucose uptake was shown to be biphasic; in mature 3T3-L1 adipocytes, treatment for up to 6 h stimulated ET_A_R-mediated glucose transporter expression and glucose uptake, whereas longer treatments suppressed glucose uptake (94). In cultured 3T3-L1 adipocytes, prolonged ET-1 treatment has also been shown to cause insulin resistance, in an ET_A_R-mediated process (95, 96), which is consistent with another study where prolonged ET-1 stimulation enhanced lipolysis in differentiated subcutaneous white adipocytes, again mediated by ET_A_R (97). Prolonged ET-1 stimulation also results in ET_B_R-mediated suppression of insulin receptor substrate-1 (IRS-1) expression and insulin-stimulated anti-lipolytic responses in differentiated visceral adipocytes (98). Taken together, these studies demonstrate the detrimental effect that long-term exposure of ET-1 has on AT, which could contribute to the development of CMS in obesity.

From a conventional perspective, ET-1 is vasoconstrictive and pro-inflammatory. There is some evidence suggesting that ET-1 induces immune cell recruitment and activation (99, 100). ET-1 can stimulate monocytes to release IL-8 and MCP-1, which recruit neutrophils and monocytes, respectively (99). ET-1 can also stimulate degranulation of mast cells to release TNFα and VEGF (100). These *in vitro* findings suggest ET-1 can induce inflammation by activating circulating monocytes or tissue-resident mast cells, which may lead to AT inflammation and fibrosis in obesity (101).

In patients with obesity, ET-1 is upregulated in AT (97, 98). Elevated ET-1 activity has also been reported in patients with type II diabetes (102). Studies support a link between higher basal ET-1 signaling activity and greater adiposity, although whether ET-1 has causal role in excess adiposity is unknown. In support of ET-1 playing a causal role in the pathophysiology of CMS, patients with CMS have an elevated arterial ET-1 level which is reported to be associated with circulating triglyceride level (103). Enhanced ET-1 signaling is also observed in overweight individuals and is preserved in individuals with obesity suggesting heightened ET-1 signaling precedes the development of obesity and its associated complications in patients with CMS (104). In contrast, body mass index has been recently reported to be negatively correlated with ET-1 expression level in omental AT (105). Differences in these findings may be due to varying influence of other co-morbidities such as vascular diseases which were excluded in the former study (104) but not the latter (105), or the complex biphasic actions of ET-1 as described above.

Although whether AT ET-1 signaling plays a causal role in obesity remains inconclusive, preclinical studies suggest inhibiting ET-1 could be another possible therapeutic strategy for CMS. Mice with endothelial-specific overexpression of ET-1 fed with chow diet displayed impaired glucose intolerance and lower systemic energy expenditure (105). However, endothelial-specific ET-1 overexpression did not attenuate Western diet-induced WAT inflammation in mice (105). Pharmacological inhibition of systemic ET-1 signaling, using either the ET_A_R-specific inhibitor atrasentan or the non-specific ET receptor inhibitor bosentan, lowered fasting blood glucose and enhanced adiponectin secretion in high fat diet-challenged mice (106). Atrasentan administration also lowered circulating triglycerides in high fat diet-challenged mice (106). The improved metabolic phenotype observed in high fat diet-challenged mice treated with of ET receptor blockers may be in part due to attenuated ET receptor-mediated perturbation in AT function (106). Besides, atrasentan administration also alleviated high fat diet-induced AT inflammation, as evidenced in reduced accumulation of immune cells and gene expression of pro-inflammatory cytokines such as TNFα (106). ET_A_R-selective inhibition may be a promising pharmacological intervention to combat perturbed metabolism and elevated inflammation in AT in CMS.

### Platelet-Derived Growth Factor-CC (PDGF-CC)

Platelet-derived Growth Factor (PDGF)-CC is a protein mediator formed by two PDGF-C monomers (107). PDGF-CC is expressed in various cell types, which share a mesenchymal origin, including the endothelium (107, 108). It is produced and secreted in its full length and digested by extracellular proteases, such as plasmin, to release its active growth factor fragment (109). Endothelial release of PDGF-CC can be upregulated by VEGF (108). The growth factor fragment acts on the transmembrane PDGF receptor-alpha (PDGFRα) to mediate various cellular processes including proliferation (109).

A study using genetically modified mice and β3-selective adrenergic stimulation, using a β3 adrenoceptor agonist, CL-316243, demonstrated that the endothelium responds to adipocyte-secreted VEGF to secrete PDGF-CC, which then induces beiging in neighboring white adipocytes (108). Although increasing PDGF-CC signaling may be explored to develop adipocyte beiging therapeutics, it may also pose a risk of inducing WAT fibrosis. Between the two identified subpopulations of PDGFRα-positive adipocyte progenitors, high fat diet activates PDGFRα-positive adipocyte progenitors with high CD9 expression, which are more proliferative and pro-fibrotic in WAT than their low CD9-expressing counterparts (110). Notably, higher levels of PDGFRα-positive high CD9-expressing adipocyte progenitors were identified in patients with obesity and glucose intolerance or diabetes (110). As PDGFRα can also be activated by PDGF-AA in WAT (110), these findings suggest caution on specificity of adipocyte progenitor subset activation is required when designing therapeutics manipulating PDGFRα signaling to treat obesity and diabetes.

### Heparanase

Heparanase is a heterodimeric endoglycosidase responsible for degrading cell surface or extracellular heparan sulfate proteoglycans (HSPGs) and is secreted by various mammalian cell types including endothelial cells (111). Heparanase is first synthesized as a 60 kDa protein, before undergoing lysosomal modification to give an active 50 kDa protein. Upon activation by cytokines, such as TNFα and IL-1beta (IL-1β) or OxLDL, endothelial cells secrete heparanase into the extracellular matrix (112). In contrast, vascular endothelial growth factor (VEGF) inhibits basal endothelial heparanase secretion (112).

Upon release into the extracellular matrix, heparanase breaks down matrix or membrane-bound HSPGs. HSPGs are heparan sulfate chains-containing glycoproteins, which function as structural proteins and cell surface receptors to mediate cellular signaling (113). HSPGs bind to a wide variety of molecules such as chemokines, growth factors and extracellular matrix proteins and enable a regulated release of such proteins upon their degradation by heparanase (111). Of interest to this review, endothelial-released heparanase mediates AT metabolism and inflammation. In terms of metabolism, endothelial cells activated by lysophosphatidylcholine, a product of lipoprotein lipase (LPL)-mediated lipolysis, secrete heparanase to modulate adipocyte secretion of LPL (114). LPL is taken up by endothelial cells and expressed at the luminal surface to hydrolyse circulating triglycerides, hence, the heparanase-HSPG signaling serves as a positive feedback loop to luminal LPL expression (114). In addition, as cell surface HSPG and HSPG-bound LPL mediate effective fatty acid uptake and storage in adipocytes (115, 116), depleting adipocyte HSPG and LPL may impair adipocyte lipid uptake and stimulate *de novo* fatty acid synthesis.

Regarding inflammation, endothelial cell-released heparanase is pro-inflammatory and pro-atherogenic. High levels of heparanase expression are found in atherosclerotic plagues in ApoE-knockout mice, suggesting heparanse may be involved in inflammation (112). As HSPGs govern immune cell recruitment and activation, and extracellular matrix composition (113, 117), elevation in heparanse-HSPG signaling in AT may drive AT inflammation and fibrosis in CMS.

Heparanase levels are significantly higher in both the plasma and the urine of humans with diabetes compared with healthy individuals (118). Higher heparanase gene expression was also found in the visceral AT of hyperglycaemic type 1 diabetic rats (119). Although the regulatory role in lipid metabolism and inflammation of heparanse-HSPG signaling has been extensively studied, the focus was mostly its effect in atherosclerosis and cancer, and not in CMS. Future work examining the AT-specific effect of the signaling pathway is needed to support the suggestion that endothelial-secreted heparanse may be involved in the progression of AT dysfunction in CMS.

### Classic Pro-inflammatory Mediators

In obesity, AT switches to a more pro-inflammatory profile, with elevated secretion of pro-inflammatory mediators such as tumor necrosis factor α (TNFα) and interleukin(IL)-6 (120–122). Alongside adipocytes, other cell types such as macrophages and endothelial cells are suggested as the primary source of pro-inflammatory mediators in the AT in obesity (120). Endothelial cells have been shown to secrete a diverse profile of pro-inflammatory mediators in response to adipocyte-derived signals (123). Twenty four-hour treatment using conditioned media from healthy human adipocytes stimulated sustained secretion of pro-inflammatory cytokines including TNFα and IL-6 from human umbilical endothelial cells (HUVECs) (123). Both TNFα and IL-6 have been extensively studied and reviewed as promoters of an inflammatory response, with local recruitment of immune cells, such as macrophages, which contribute to chronic inflammation, and may in turn lead to insulin resistance in AT (122). In addition to their pro-inflammatory actions, these endothelial-derived cytokines can also act directly on adipocytes to mediate various metabolic functions.

#### Tumor Necrosis Factor-Alpha (TNFα)

TNFα is a member of the TNF family, known to be primarily secreted by monocytes or macrophages (124). TNFα can also be synthesized and secreted by endothelial cells upon stimulation by pro-inflammatory signals such as a combination of interferon-γ, IL-1β and lipopolysaccharide (125), or IL-1α (126). TNFα signals via binding to TNF receptor (TNFR)-1 or TNFR-2 of the TNFR superfamily, thus activating its downstream signaling cascades involving protein partners such as c-Jun N-terminal kinases (JNK) and mitogen-activated protein kinase (MAPK) (124). In adipocytes, TNFα induces insulin resistance and upregulates lipolysis (127, 128). Prolonged exposure to exogenous TNFα for 24 or 72 h inhibited insulin-stimulated glucose uptake and induced glycerol release in cultured primary human adipocytes (127). On a molecular level, 3-day TNFα treatment inhibited insulin-stimulated insulin receptor (IR) and IRS-1 phosphorylation in murine 3T3-F442A adipocytes (128). Consistent with these findings using cultured adipocytes, neutralizing TNFα activity by application of a soluble TNF receptor-IgG protein improved insulin-stimulated IR and IRS-1 phosphorylation in AT and lowered circulating free fatty acid concentration in genetically obese Zucker rats (129). In addition, high fat diet-challenged TNFα-deficient mice had lower circulating free fatty acids and fasting insulin levels compared to control littermates, suggesting suppressing TNFα activity attenuated unfavorable lipolysis and hyperinsulinemia in diet-induced obesity (5). In line with the finding that TNFα production and secretion is upregulated in AT from human patients with obesity (130), these studies highlight the therapeutic potential of targeting TNFα to combat dyslipidemia and insulin resistance in CMS. Unfortunately, current clinical trials have reported conflicting outcomes. Increased adiposity or body weight gain were reported as side effects in a study treating obesity or autoimmune diseases with anti-TNFα antibodies (131). In contrast, the TNFα inhibitor etanercept was shown to lower fasting glucose in patients with obesity over a 6-month period (132). These conflicting findings highlight the need for an alternative therapeutic target of higher specificity in downstream TNFα signaling in adipocytes, or refinement of treatment regimes. Future studies should also determine the effect of targeting TNFα on inflammation or susceptibility to infection in treated patients, as increased risk of infections has been reported in some patients receiving anti-TNF therapy (133).

#### Interleukin-6 (IL-6)

Similar to TNFα, IL-6 can be synthesized and secreted by endothelial cells upon stimulation by pro-inflammatory cytokines such as IL-1α, and interestingly, TNFα (134). IL-6 signals via binding to a transmembrane receptor complex consisting of a IL-6 receptor subunit and two signal transducing receptor gp130 subunits (135). The activated receptor complex conducts downstream signaling, mediated by signal transducer and activator of transcription (STAT) proteins or mitogen-activated protein kinase (MAPK) (135). In adipocytes, similar to TNFα, prolonged exposure to IL-6 induces insulin resistance and upregulates lipolysis (136, 137). Prolonged IL-6 stimulation enhanced glucose uptake (138), but induced insulin resistance by inhibiting expression of IRS-1, in cultured murine 3T3-L1 adipocytes (136). IL-6 also stimulated glycerol secretion of subcutaneous and omental AT cultures isolated from human donors with obesity *in vitro*, suggesting IL-6 stimulates basal lipolysis in adipocytes (137). In agreement with these findings, isolated mesenteric adipocytes from high fat diet-challenged obese adipocyte-specific gp130-knockout mice, possess a lower rate of basal lipolysis and leptin secretion compared to isolated adipocytes from control littermates (139). However, it is of note that these mice also demonstrated impaired glucose tolerance, associated with suppressed adipose leptin secretion and intestinal glucagon-like peptide-1 (GLP-1) release, suggesting that adipose IL-6 signaling is important in mediating glucose metabolism *in vivo* (140). In humans, circulating IL-6 levels are positively correlated with body mass index and percent body fat (141, 142). We have previously reported a negative correlation between serum IL-6 and endothelial-dependent vasodilatation in humans across a range of BMIs (143). In addition, elevated serum IL-6 levels were found in individuals with impaired glucose tolerance and type 2 diabetes (T2DM) (144, 145). Hence, in obesity, endothelial cells activated by pro-inflammatory cytokine-activated may contribute to the already elevated serum IL-6 level by secreting more IL-6, as part of a positive feedback loop. Suppressing IL-6 signaling is shown to improve hepatic insulin sensitivity in obese mice (139). As IL-6 also plays a key role in mediating skeletal muscle glucose disposal and pancreatic insulin secretion (146), future research into targeting downstream IL-6 signaling with greater tissue specificity should be undertaken to develop anti-diabetic treatments of high efficacy.

## Small Molecules

### Reactive Oxygen Species (ROS)

Reactive oxygen species (ROS) are highly reactive intermediates of oxygen produced by a range of cellular enzymes, such as NADPH oxidases (NOXs), complexes I and III of the mitochondrial respiratory chain, uncoupled eNOS or xanthine oxidases (XOs) (147). Most of the reactions mediated by these enzymes generate superoxide (${\text{O}}_{2}^{-}$), which has a very short half-life and is converted to hydrogen peroxide (H_2_O_2_) and peroxynitrite (ONOO^−^) after reacting with water or NO, respectively. Under normal physiological conditions, intracellular ROS mediate cellular processes such as proliferation and differentiation (148). However, in diseased states, such as insulin resistance where eNOS is uncoupled and generates a large amount of superoxide, which can become stress signals, resulting in increased tissue or vascular inflammation and downregulated adipocyte function (147, 149, 150).

#### Hydrogen Peroxides (H_2_O_2_)

In the endothelium, H_2_O_2_ is generated via a number of pathways, including dismutation of ${\text{O}}_{2}^{-}$ spontaneously or catalyzed by superoxide dismutase (SOD) or direct production by XOs and glucose oxidases (151). It has been reported that physiological concentration of H_2_O_2_ in human blood is approximately 10 μM (152). H_2_O_2_ mediates cellular processes such as cell proliferation and energy metabolism by interacting with different signaling partners including adenosine monophosphate-activated protein kinase (AMPK) (153, 154). Depending on the site of production and the availability of aquaporins to facilitate cross-membrane diffusion, H_2_O_2_ can act both intracellularly and intercellularly (154, 155).

Under normal physiological conditions, H_2_O_2_ regulates endothelial secretion of vascular endothelial growth factor (VEGF) and eNOS activity (151). Extracellular H_2_O_2_ may modulate adipocyte function, H_2_O_2_ has been shown to enhance murine 3T3-L1 adipocyte differentiation (156). At high concentrations (400 μM), H_2_O_2_ increased conversion of glucose to glycogen by activating glycogen synthase in cultured rat adipocytes (157). H_2_O_2_ has also been shown to induce basal lipolysis but inhibit adrenergic or glucagon-stimulated lipolysis in rat adipocytes (158). These studies highlight the potential (albeit complex) role of H_2_O_2_ in modulating adipocyte metabolism.

H_2_O_2_ is also known to influence vascular tone and inflammation. Depending on the concentration, vascular bed or presence of other vasoactive substances, H_2_O_2_ can act as a vasodilator (159, 160) or vasoconstrictor (161, 162). H_2_O_2_ also acts as a pro-inflammatory mediator, high concentrations of H_2_O_2_ promote leukocyte recruitment by upregulating endothelial expression of leukocyte adhesion molecules (163). H_2_O_2_ has also been shown to induce gene expression of pro-inflammatory mediators such as IL-6 and MCP-1 in a dose-dependent manner in cultured 3T3-L1 adipocytes (164). These findings suggests that extracellular H_2_O_2_ switches endothelial cells and adipocytes to possess a more pro-inflammatory phenotype.

In patients with obesity, higher levels of H_2_O_2_ were determined in visceral AT and were suggested to correlate with insulin resistance (165) as a result of upregulated endothelial release of H_2_O_2_. In diet-induced obese mice, endothelial extracellular H_2_O_2_ production is upregulated (166), predominantly by NOX4, and is suggested to mediate glucose disposal in BAT (167). We recently reported that endothelial insulin and IGF-1 signaling governs endothelial production of miR-25 which suppresses NOX4 expression (167). Endothelial-specific expression of mutant IGF-1R receptors which form non-functioning hybrid receptors with insulin receptors and IGF-1R improved insulin-stimulated glucose uptake in brown AT and muscle in mice, in a H_2_O_2_ –dependent manner (167). Our findings suggest that blunted endothelial NOX4 activity and subsequent blunted endothelial production of H_2_O_2_ may contribute to impaired brown adipocyte glucose uptake and development of insulin-resistance in obesity (167).

Despite evidence suggesting some favorable metabolic effects modulated by H_2_O_2_, high concentrations of H_2_O_2_ are unlikely to be therapeutic. It is important to note that H_2_O_2_ can also be generated by NOXs in insulin-stimulated adipocytes or by mitochondrial respiration complexes in stressed adipocytes (168, 169). *In vitro* evidence has suggested that high intracellular levels of ROS and prolonged hyperinsulinaemia can induce apoptosis in brown adipocytes (170). Accumulation of mitochondrial-generated ROS (including H_2_O_2_) is also reported to induce insulin resistance and suppress adiponectin secretion in adipocytes (169). This raises the question of whether H_2_O_2_ released from different cellular sources (endothelial cells or adipocytes) or under different circumstances (normal or pathological conditions) may produce different outcomes in mediating adipocyte health and function.

Aiming to counteract H_2_O_2_-mediated ill-effects in adipose function, some studies have proposed suppression of H_2_O_2_ signaling as a potential therapeutic option in CMS. Treatment with antioxidant NAC (N-acetylcysteine) reversed H_2_O_2_-suppressed adiponectin secretion in mature 3T3-L1 adipocytes (164). Treatment with another antioxidant apocynin reduced WAT level of H_2_O_2_, enhanced plasma adiponectin levels, reduced glucose and insulin levels and reduced pro-inflammatory TNFα gene expression in obese KKAy mice, suggesting suppression of H_2_O_2_ production has favorable metabolic effects and may alleviate WAT inflammation in obesity *in vivo* (164). Similar therapeutic benefits were observed in apocynin-treated high fat diet-challenged obese C57BL/6 mice (171).

#### Peroxynitrite (ONOO^–^)

Obesity, and diabetes-related stress signals such as cytokines and hyperglycaemia, stimulate the production of a large amount of peroxynitrite (ONOO^−^) as superoxide react with the NO generated by enzymes such as NOXs, uncoupled NO and iNOS (59, 172). ONOO^−^ can react with different molecules such as carbon dioxide to produce reactive nitrogen species or hydroxyl radicals (59). ONOO^−^ and its derivatives either diffuse or are transported via anion channels across the plasma membrane, initiating downstream signaling in neighboring cells (172). ONOO^−^ can react with a wide range of cellular targets, including prostacyclin synthase (173), low-density lipoproteins (174) and DNA (175), making it a very powerful inducer of unfavorable metabolic and pro-inflammatory effects.

In AT, ONOO^−^ induces insulin resistance and cell death. In the submillimolar range, ONOO^−^ increased glucose uptake dose-dependently, whereas at higher concentrations (millimolar range), it reduced viability of cultured 3T3-L1 adipocytes (176). Treatment using higher concentrations of an ONOO^−^ donor (in millimolar range) also inhibited insulin-stimulated glucose uptake by tyrosine nitration and subsequent inhibition of insulin receptor substrate-1 (IRS-1) in cultured 3T3-L1 adipocytes (177). ONOO^−^ can also cause nitration of various proteins, which control energy homeostasis including fatty acid binding protein-4 (FABP-4) in adipocytes (178). As an example, nitrated FABP-4 has reduced binding affinity to lipids (178), which may result in dysregulated lipid release in adipocytes. As FABP-4 plays an important role in modulating lipid release and metabolism in adipocytes via its interaction with hormone-sensitive lipase (179) or PPARγ transcription factors (180), peroxynitrite-mediated nitration of FABP-4 may contribute to the development of dysregulated lipolysis and insulin resistance in adipocytes in obesity.

In addition to inducing insulin resistance, peroxynitrite is known as a pro-inflammatory mediator and is suggested to play a role in driving vascular diseases (59). Of relevance to AT, peroxynitrite may lead to activation of pro-inflammatory signaling cascades in endothelial cells and circulating immune cells, resulting in adipose inflammation. In bovine microvascular endothelial cells, treatment with an ONOO^−^ donor elevated iNOS and NFκB protein expression, but suppressed prostacyclin synthase expression, suggesting an increase in iNOS and NFκB pro-inflammatory signaling but a decrease in anti-inflammatory vasodilator prostacyclin production (181). As NFκB signaling modulates endothelial activation and secretion of pro-inflammatory cytokines (reviewed in 3.4 above), peroxynitrite may contribute to adipose inflammation by enhancing recruitment and activation of immune cells. Moreover, peroxynitrite can activate leukocytes to release IL-8 (182), which attracts neutrophils to sites of inflammation (183). Lastly, peroxynitrite-induced apoptotic adipocytes may also themselves serve as pro-inflammatory signals, and drive tissue inflammation (184).

Despite some evidence from *in vitro* reports that targeting peroxynitrite may improve AT health and function, studies using systemic intervention seems to suggest otherwise. Global iNOS-knockout mice demonstrated improved glucose tolerance and insulin sensitivity, but the effect was likely limited to skeletal muscle rather than AT (185). More importantly, abrogating ROS using long-term antioxidant supplementation did not seem to lower the risk of developing CMS in humans despite a negative association between serum antioxidant concentration and risk of CMS (186). These findings suggest that perhaps ROS play a smaller role in driving AT dysfunction in CMS and that targeting ROS may not be sufficient to reverse severe metabolic defects and chronic inflammation in humans.

### Prostaglandins

Prostaglandins are a family of lipid mediators formed from the conversion of arachidonic acids catalyzed by respective synthases (187). In AT, prostaglandin I2 (PGI_2_) and E2 (PGE_2_) are the two most abundant members of the family present (188). Studies have proposed the importance of the presence of both non-fat cells, predominantly endothelial cells, and adipocytes for optimal AT prostaglandin production and secretion (189, 190). Experiments using isolated endothelial cells from human adipose capillaries presented evidences that human AT endothelial cells are capable to secrete both PGI_2_ and PGE_2_ (191).

#### Prostacyclin (PGI_2_)

Prostacyclin (also called prostaglandin I2 or PGI_2_) inhibits platelet activation and is also a vasodilator (192). PGI_2_ is the most dominant prostanoid produced in the endothelium (193). PGI_2_ is generated by prostacyclin synthase, which uses prostaglandin endoperoxides converted from arachidonic acid by cyclooxygenases (194). The synthesis of PGI_2_ takes place only upon endothelial cell activation by substances such as thrombin (195), endothelins (196, 197), platelet-derived microparticles (198), angiotensin I (199) or mechanically by shear stress (200). In particular to the AT, endothelium is the main source of PGI_2_ by utilizing adipocyte-released arachidonic acid (189).

Endothelial-derived PGI_2_ acts on the prostacyclin receptor (IP) and may influence adipogenesis and adipocyte beiging via both IP-dependent and IP-independent pathways (201). It is reported that IP gene expression is upregulated during adipogenesis, and its activation induces beiging of white adipocytes in culture (201, 202). Treatment using carbaprostacyclin, a PGI_2_ analog, induced gene transcription of UCP-1 and peroxisome proliferator-activated receptor gamma coactivator 1-alpha (PGC1α) in primary stromal vascular fraction cell cultures isolated from murine white AT, suggesting a role of PGI_2_ in activating adipocyte precursors to form energy-expending beige adipocytes by upregulating mitochondrial uncoupled respiration and biogenesis (203). Carbaprostacyclin also increased gene expression of beige adipocyte markers in mature human adipocytes (202). Taken together, these studies suggest that endothelial-derived PGI_2_ contributes to the activation of preadipocytes, giving rise to energy-expending beige adipocytes.

Similar to NO, PGI_2_ is an anti-inflammatory signaling molecule. Pharmacological activation of IP results in a reduction of cytokine-stimulated expression of cell adhesion molecules and immune cell recruitment in endothelial cells *in vitro* (204, 205). Treatment with a PGI_2_ analog iloprost attenuates lymphocyte adhesion and reduces IL-1 stimulated expression of ICAM-1 and E-selectin in HUVECs (204). Treatment using another PGI_2_ analog beroprost sodium (BPS) reduces VCAM-1 and E-selectin expression in TNFα-stimulated HUVECs (205). It is worth noting that the anti-inflammatory effects of PGI_2_ were not observed in unstimulated endothelial cells in either study (204, 205), suggesting PGI_2_ is important in suppressing inflammation only in activated endothelial cells.

In both genetic induced obesity in rats, and high fat diet-induced obese mice, PGI_2_ synthase activity is impaired (206); subsequent administration of PGI_2_ was shown to improve metabolic profiles in both of these models (207, 208). Oral treatment with BPS improved glucose tolerance and insulin sensitivity, and attenuated WAT inflammation in high fat diet-challenged obese mice (208). These animal studies suggest increasing PGI_2_ signaling could lead to beneficial metabolic and anti-inflammatory outcomes.

However, the translational potential of PGI_2_ application in activating beige AT activity has not been established successfully in human subjects. Twelve-week oral administration of BPS improved insulin sensitivity without altering body weight, BMI, waist circumference or lipid profile in human patients with type 2 diabetes (209), suggesting PGI_2_ supplementation improves insulin sensitivity without improving AT function. In contrast, another study using 3-year administration of BPS reported no improvement in insulin sensitivity in patients with type 2 diabetes (205). Although IP activation may alleviate inflammation in patients with T2DM (205), it is yet to be verified whether IP activation improves AT function and systemic homeostasis in patients with CMS.

#### Prostaglandin E2 (PGE_2_)

Prostaglandin E2 (PGE_2_) can be produced by prostaglandin E (PDE) synthase-catalyzed derivatization of arachidonic acid in adipocytes (210), preadipocytes (211) and endothelial cells (191). In endothelial cells, PGE_2_ production can be upregulated by stimuli including histamine (212) and angiotensin I (199). Secreted PGE_2_ exerts downstream signaling effects via PGE receptors subtype 1–4 (EP1–4) (187). Depending on the subtype of PGE receptors, PGE_2_ can elicit different metabolic outcomes in recipient cells. Of interest to this review, EP3 and EP4 actions are reviewed below as these two subtypes are better studied.

In terms of metabolism, EP3 suppresses preadipocyte differentiation and lipolysis in adipocytes. EP3 antagonism by L-798106 enhanced adipogenesis in cultured mouse embryonic fibroblasts (213), suggesting EP3 activation suppresses preadipocyte differentiation. In line of agreement, isolated preadipocytes from EP3-deficient mice displayed a higher differentiation potential (213). In addition, EP3-deficient mice possessed higher circulating glycerol level and cultured adipocytes from EP3-deficient mice exhibited higher rate of lipolysis, suggesting EP3 suppresses lipolysis (213).

In contrast, EP4 activation induces lipolysis and insulin resistance. Pharmacological EP4-specific activation by ONO-AE1-329 increased adipose triglyceride lipase-encoding Pnpla2 gene expression and hormone-sensitive lipase phosphorylation and inhibited insulin-stimulated Akt phosphorylation in cultured OP9 adipocytes, suggesting EP4 activation can increase lipolysis and insulin resistance (214). In line of agreement, adipocyte-specific EP4-deficient mice displayed increased body weight, reduced WAT Pnpla2 gene expression and lower circulating free fatty acids under chow diet (214). In addition, it was reported that PGE_2_ induced leptin secretion in subcutaneous adipose tissue isolated from human donors with obesity (215) and increased beiging potential of murine preadipocytes (216).

Similarly to PGI_2_, PGE_2_ exerts anti-inflammatory effects. Pretreatment using PGE_2_ activated EP4 and suppressed production of pro-inflammatory cytokines such as IL-8, MCP-1, macrophage inflammatory protein-1beta (MIP1β) and interferon gamma-induced protein-10 (IP-10) in lipopolysaccharides (LPS)-activated human macrophages (217). In the same report, MIP1β and MCP-1 production in human macrophages activated by obesity-related stress signal TNFα was also attenuated by PGE_2_ (212). Similar anti-inflammatory effects were reported in mice studies (218). Pretreatment using PGE_2_ reduced pro-inflammatory MIP1β and IP-10 production in isolated murine adipocytes as well as control but not EP4-deficient LPS-activated mouse WAT explants (218). Taken together, these reports support the notion that PGE_2_-EP4 signaling suppresses adipocyte and macrophage inflammatory response upon activation.

In murine models of diet-induced obesity, higher PGE_2_ and EP4 levels (218) but lower EP3 level (213) were reported in AT. EP4-deficient mice were more susceptible to high fat diet-induced weight gain and adipocyte hypertrophy (214). Higher AT PGE_2_ level was also reported in humans with obesity (219). Considering the differences in subtype-mediated metabolic outcomes, changes in abundance of receptor subtypes in AT may reflect maladaptive response to overnutrition, as AT may be more sensitive to EP4-mediated lipolysis and insulin resistance and less sensitive to EP3-mediated suppression of lipolysis. Inhibiting EP4-mediated signaling may be a possible therapeutic to treat CMS. However, pharmacological studies activating EP4 seems to suggest the otherwise. Administration of an EP4 agonist, ONO-AE1-329, was reported to elicit beneficial metabolic outcomes and anti-inflammatory effects in db/db mice, as characterized by improved glucose tolerance and insulin sensitivity, and shifted AT macrophages toward anti-inflammatory M2 type (220). Administration of another EP4 agonist, CAY10580, reduced AT IP-10 and MIP-1α levels in high fat diet-challenged mice, suggesting systemic EP4 activation alleviates obesity-associated AT inflammation and such anti-inflammatory effects outweigh potential EP4-mediated metabolic defects (218). Of high therapeutic interest, non-specific activation of PGE_2_ signaling posed anti-fibrotic and anti-inflammatory effects, induced beiging and suppressed adrenergic-induced lipolysis in isolated human AT explants (219). As the favorable outcomes in this study may be partly contributed by EP1 and EP2 signaling, this finding highlights the need of future studies studying EP1 and EP2 signaling in different cell types in AT to better establish the therapeutic potential of manipulating PGE_2_ signaling as a treatment goal for CMS.

### Peroxisome Proliferator-Activated Receptor Gamma (PPARγ)-Activating Lipids

Upon activation by fatty acids, human AT endothelial cells (hATECs) increase expression of fatty acid transporters including FABP-4 to enhance fatty acid uptake from the circulation (221). hATECs then release fatty acids to adipocytes for storage (221). More importantly, endothelial cells release lipids to activate peroxisome proliferator-activated receptor gamma (PPARγ) signaling in adipocytes (221). Treatment using the lipid fraction of conditioned media from isolated hATECs was able to induce PPARγ activation in primary human adipocytes. PPARγ activation was enhanced further when the hATECs were pre-treated with oleic acid, a fatty acid (221). Human adipocytes are unable to produce PPARγ agonists but are responsive to PPARγ stimuli (221), therefore, these findings demonstrated that the AT endothelial cells are able to respond to circulating fatty acids, and secrete lipids, activating PPARγ signaling in adipocytes. As PPARγ is a key regulator of adipogenesis and adipocyte function, including mitochondrial respiration (222), future studies examining the identity of endothelial-derived PPARγ ligand and elucidating the mechanism by which the activated endothelial cells secrete such ligands may give rise to an alternative drug candidate to combat diseased adiposity in obesity.

## Concluding Remarks

Balanced coordination of metabolic processes in the adipose tissue, liver, skeletal muscle and pancreas are crucial to whole-body energy homeostasis. Dysfunction of these highly metabolic organs can lead to the development of the cardiometabolic syndrome. In the past few decades, a considerable amount of interest has been generated in exploiting inter-organ signaling pathways, particularly organ-specific endocrine factors such as adipose-derived adiponectin, and liver-derived fibroblast growth factor 21, to treat metabolic disorders (223–225). However, the localized crosstalk between the heterogeneous endothelium and these metabolic organs is still yet to be fully explored.

AT is crucial in mediating systemic homeostasis. Along with governing energy level by regulating glucose and lipid metabolism, AT secretes adipokines such as adiponectin and leptin which can act on distal organs to modulate insulin sensitivity and food intake. The AT is a sophisticated organ made up of adipocytes, supported by stromal vascular cells composed of endothelial cells, fibroblasts, pericytes, immune cells and adipose progenitors (226). Among different types of stromal vascular cells, endothelial cells serve as the first responder to fluctuations in circulating signaling cues such as hormones or glucose and AT-derived signals such as pro-inflammatory mediators and lipids. Endothelial dysfunction in AT could drive different components of AT dysfunction such as dyslipidaemia, increased fibrosis, chronic inflammation and insulin resistance. It is worth noted that functional heterogeneity exists among different AT depots (227). This review focuses on the role of endothelial-derived mediators in AT depots at classic locations such as subcutaneous and omental AT due to their significance in mediating whole-body energy homeostasis and contribution to the progression of obesity. Recent studies have shed light on the importance of perivascular AT in regulating local energy homeostasis around blood vessels and vital organs such as the heart and the disastrous vascular complications caused by perivascular AT dysfunction (228–230). Future work dissecting the role of endothelial-derived factors in mediating perivascular AT function specifically may provide new insights in our understanding of the paracrine role of endothelium in this depot and its contribution to the vascular components of CMS (231).

Over the past decade, our understanding of the endothelium has evolved, from an inert barrier layer in the vasculature to an active and diverse metabolic organ. Following advances in biological techniques such as multi-omics, it has become possible to separate and identify individual endothelial-derived metabolites, both from different subsets of endothelium, and across species. This deepens our understanding of the role of the endothelium as an active organ, orchestrating the function of neighboring tissue (and of interest to this review, the AT), as well as expanding our knowledge on the consequences of endothelial dysfunction in metabolic and cardiovascular diseases.

**Figure 1:**
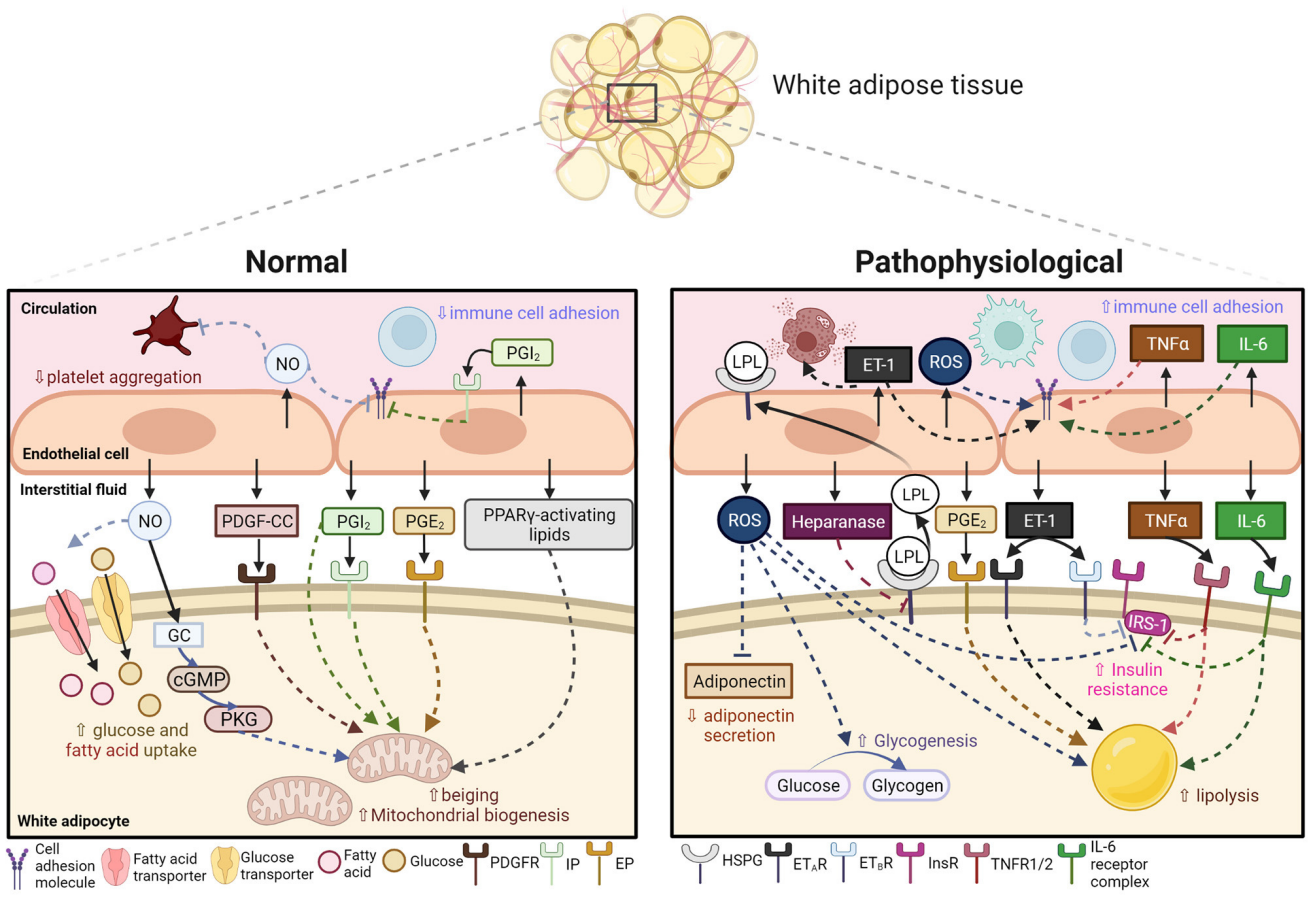
Endothelial-derived signals in mediating AT metabolism and inflammation under normal and pathophysiological conditions in CMS. Under normal conditions, the adipose endothelium releases nitric oxide (NO), platelet-derived growth factor-CC (PDGF-CC), prostacyclin (PGI_2_), prostaglandin E2 (PGE_2_) and peroxisome proliferator-activated receptor gamma (PPARγ)-activating lipid in response to changes in microenvironment. These biological signals then act on downstream signaling molecules (soluble guanylate cyclase, GC; cyclic guanine monophosphate, cGMP; protein kinase G, PKG) or receptors (platelet-derived growth factor receptor, PDGFR; prostacyclin receptor, IP; prostaglandin E2 receptor, EP) in neighboring white adipocytes, resulting in increased glucose and fatty acid uptake and upregulated beiging and mitochondrial biogenesis. NO and PGI_2_ also act on the endothelium and circulating platelets to suppress immune cell adhesion and platelet aggregation. Under pathophysiological conditions, circulating or adipose-derived pro-inflammatory signals stimulate the endothelium to release reactive oxygen species (ROS), heparanase, endothelin-1 (ET-1), tumor necrosis factor-alpha (TNFα) and interleukin-6 (IL-6). These signals act on downstream signaling partners (heparan sulfate proteoglycans, HSPG; endothelin receptors type A and B, ET_A_R and ET_B_R; TNF receptor 1/2, TNFR1/2; IL-6 receptor complex) to inhibit adiponectin secretion, increase endothelial expression of lipoprotein lipase (LPL), upregulate glycogenesis and lipolysis, and induce insulin resistance by inhibiting insulin receptor (InsR)-insulin receptor substrate-1 (IRS-1) signaling in white adipocytes. These signals also pose pro-inflammatory effects by increasing recruitment and activation of immune cells. Created with BioRender.com.

Here we have presented an overview of various key endothelial-derived regulatory factors which induce local effects on the metabolism and inflammation of the AT and discussed potential areas of future development on exploiting these pathways to treat cardiometabolic syndrome (Table 1; Figure 1). Among various regulatory factors reviewed here, NO, PGI_2_, PGE_2_, PDGF-CC, and the yet-to-be-identified PPARγ-activating lipid, share great therapeutic potential to upregulate energy expenditure by increasing adipocyte beiging, enhancing AT insulin sensitivity, and alleviating undesirable AT inflammation. Our recent report also suggested that endothelial-derived ROS plays a role in enhancing whole-body (including BAT) insulin sensitivity in response to short-term overnutrition (167). However, diseased upregulation of endothelial-derived ET-1, ROS, heparanse and pro-inflammatory mediators can bring detrimental metabolic and inflammatory outcomes by upregulating lipolysis and inducing insulin resistance. It is worth noting that, as some of these regulatory factors (such as NO, PGI_2_ and ET-1) also play a role in mediating vascular tone and inflammation, the vascular effects of targeting these pathways must also be considered during drug development. For instance, reduced NO bioavailability (232) and ET-1 hyperactivity (233) have been reported in patients with hypertension (233), one of the components of CMS. Exploiting these biologics may bring beneficial therapeutic outcomes to both the metabolic and vascular components of cardiometabolic syndrome.

**Table 1:** Metabolic and inflammatory actions of endothelial-derived signals in AT under normal and pathophysiological conditions in CMS.

**Endothelial-derived signal**	**Metabolic action**	**Inflammatory action**
Nitric oxide (NO)	• Enhances mitochondrial biogenesis in adipocytes (69). • Induces beiging in adipocytes (70, 71, 73) • Upregulates mitochondrial respiration and increases uptake and oxidation of fatty acid and glucose in adipocytes (71, 72)	• Inhibits platelet aggregation (74) • Suppresses endothelial expression of adhesion molecules (75)
Endothelin-1 (ET-1)	• Short-term exposure increases glucose uptake (94) • Long-term exposure decreases glucose uptake in adipocytes (94) • Long-term exposure also causes insulin resistance (95, 96, 98) and induces lipolysis (93, 97).	• Stimulates monocytes to release IL-8 and MCP-1, which recruit neutrophils and monocytes, respectively (99) • Stimulates degranulation of mast cells to release TNFα and VEGF (100)
Platelet-derived Growth Factor-CC (PDGF-CC)	• Induces beiging in adipocytes (108)	
Heparanse	• Increases adipocyte secretion of lipoprotein lipase (LPL) (114)	• Increases immune cell recruitment and activation, and alters extracellular matrix composition (113, 117)
Tumor necrosis factor-alpha (TNFα)	• Induces insulin resistance and upregulates lipolysis in adipocytes (127, 128).	• Increases local recruitment and activation of immune cells (122)
Interleukin-6 (IL-6)	• Induces insulin resistance and upregulates lipolysis in adipocytes (136, 137).	• Increases local recruitment and activation of immune cells (122)
Hydrogen peroxide (H_2_O_2_)	• Enhances murine 3T3-L1 adipocyte differentiation (156) • At high concentrations, H_2_O_2_ increased conversion of glucose to glycogen in adipocytes (157); • Induces basal lipolysis but inhibit adrenergic or glucagon-stimulated lipolysis in adipocytes (158)	• At high concentration, H_2_O_2_ promotes leukocyte recruitment (163) • Induces gene expression of pro-inflammatory mediators in adipocytes (164)
Peroxynitrite (ONOO^−^)	• In the submillimolar range, ONOO^−^ increases glucose uptake in adipocytes (176) • At higher concentrations, it causes apoptosis of adipocytes (176); • And induces insulin resistance in adipocytes (177) • Can also cause dysregulated lipolysis (178–180)	• Induces endothelial activation and secretion of pro-inflammatory cytokines, promoting recruitment and activation of immune cells (181) • Directly activates leukocytes to release IL-8 (182), which attracts neutrophils to sites of inflammation (183)
Prostacylin (PGI_2_)	• Induces beiging of white adipocytes in culture (201, 202) • Upregulates mitochondrial uncoupled respiration and biogenesis in adipocytes (203)	• Suppresses expression of cell adhesion molecules and immune cell recruitment in activated endothelial cells (204, 205)
Prostaglandin E2 (PGE_2_)	• Suppresses preadipocyte differentiation and lipolysis in adipocytes by activating PGE receptor subtype 3 (213) • Can induce induces lipolysis and insulin resistance in adipocytes by activating PGE receptor subtype 4 (214) • Induces leptin secretion by adipocytes (215)	• Suppresses EP4-mediated production of pro-inflammatory cytokines in activated macrophages (217) and adipocytes (218)
Peroxisome proliferator-activated receptor gamma (PPARγ)-activating lipids	• Activates PPARγ signaling (221), which governs adipogenesis and function of adipocytes (222)	

In the future, several key areas of research include point (1) exploring further the role of endothelial-derived small molecules and microribonucleic acids (miRNAs) as well as other proteins as local regulators of metabolism in AT and other metabolic organs; (2) investigating the heterogeneity of the endothelium due to differences in environment (tissue), species (rodents or human) and model (cell-lines or primary cells, healthy or diseased models); (3) revisiting current literature on developing therapeutics and evaluating both metabolic and vascular outcomes of such. To conclude, we believe that current understanding on endothelial crosstalk with neighboring cells in metabolic organs will remain a hot topic, and foster the development of therapeutics using biologics to combat metabolic disorders.

## Author Contributions

CL performed literature search and wrote this manuscript. NH, KB, and MK reviewed this manuscript. All authors contributed to the article and approved the submitted version.

## Funding

CL was funded by a British Heart Foundation studentship (FS/19/59/34896). NH was funded by British Heart Foundation Project grant (PG/18/82/34120). MK holds a British Heart Foundation Chair in Cardiovascular and Diabetes Research (RG/15/7/31521).

## Conflict of Interest

The authors declare that the research was conducted in the absence of any commercial or financial relationships that could be construed as a potential conflict of interest.

## Publisher's Note

All claims expressed in this article are solely those of the authors and do not necessarily represent those of their affiliated organizations, or those of the publisher, the editors and the reviewers. Any product that may be evaluated in this article, or claim that may be made by its manufacturer, is not guaranteed or endorsed by the publisher.
